# Heat shock factor binding protein *BrHSBP1* regulates seed and pod development in *Brassica rapa*

**DOI:** 10.3389/fpls.2023.1232736

**Published:** 2023-08-30

**Authors:** Muthusamy Muthusamy, Seungmin Son, Sang Ryeol Park, Soo In Lee

**Affiliations:** Department of Agricultural Biotechnology, National Institute of Agricultural Sciences (NAS), Rural Development Administration, Jeonju, Republic of Korea

**Keywords:** CRISPR-Cas, *Brassica rapa*, floral genes, seed, *BrHBSP1*, heat stress, drought

## Abstract

Plant heat shock factor binding proteins (HSBPs) are well known for their implication in the negative regulation of heat stress response (HSR) pathways. Herein, we report on the hitherto unknown functions of *HSBP1* in *Brassica rapa (BrHSBP1)*. *BrHBSP1* was found to be predominant in flower buds and young leaves, while its segmental duplicate, *BrHSBP1-like*, was abundant in green siliques. Exposure to abiotic stress conditions, such as heat, drought, cold, and H_2_O_2_, and to phytohormones was found to differentially regulate *BrHSBP1*. The activity of BrHSBP1-GFP fusion proteins revealed their cellular localization in nuclei and cytosols. Transgenic overexpression of *BrHSBP1* (BrHSBP1^OX^) improved pod and seed sizes, while CRISPR-Cas *BrHSBP1* knock-out mutants (*Brhsbp1_KO*) were associated with aborted seed and pod development. The transcriptomic signatures of BrHSBP1^OX^ and *Brhsbp1_KO* lines revealed that 360 and 2381 genes, respectively, were differentially expressed (Log2FC≥2, p_adj_<0.05) expressed relative to control lines. In particular, developmental processes, including plant reproductive structure development (RSD)-related genes, were relatively downregulated in *Brhsbp1_KO.* Furthermore, yeast two-hybrid assays confirmed that BrHSBP1 can physically bind to RSD and other genes. Taking the findings together, it is clear that BrHSBP1 is involved in seed development via the modulation of RSD genes. Our findings represent the addition of a new regulatory player in seed and pod development in *B. rapa*.

## 1 Introduction

Plant heat shock factor binding proteins (HSBPs) are highly conserved, small molecular weight proteins that have long been recognized for their negative regulation of the heat stress response (HSR) pathway (Hsu et al., 2010; Marko et al., 2019). In general, plants respond to abiotic stresses such as high temperature, salinity, and drought through developmental, physiological, and biochemical adjustments. These responses require the expression of stress-responsive genes, including heat stress transcription factors (HSFs) (Hsu et al., 2010). Plant HSFs are the terminal components of a signal transduction chain that mediates the expression of genes responsive to various abiotic stresses. Under stress conditions, HSFs interact with other putative stress sensors, such as heat shock protein (*HSP*) genes and heat shock factor binding protein (*HSBP*) genes, through their respective stress-responsive cis-elements (DRE/HSE) (Xiang et al., 2018). HSPs confer tolerance to drought, heat, and salinity stress by preventing aggregation and promoting the renaturation of stress-damaged, misfolded, or denatured proteins (Fragkostefanakis et al., 2015). These proteins are called molecular chaperones and are tightly regulated by HSFs (Tian et al., 2021). During heat stress, HSFs bind to the heat stress-responsive cis-elements (HSEs) of *HSP* gene promoters (Guo et al., 2020). However, HSF-mediated stress tolerance depends on the regulatory roles of plant HSBPs. HSBPs negatively affect the DNA-binding capacity and transactivation activity of HSFs (Hsu et al., 2010; Scharf et al., 2012; Guo et al., 2016), which often causes adverse cellular conditions during exposure to different abiotic stress conditions. As a first line of evidence, the loss of functions of *AtHSBP1* has been found to significantly increase the expression of HSPs, which might be essential for plant adaptation to stress. Similarly, *hsbp1* have been found to confer thermotolerance in tomato plants (Marko et al., 2019).

Plants can respond to stressors by evolving various biochemical pathways, the products of which can mitigate the deleterious effects of stress. Raffinose family oligosaccharides play a role in promoting abiotic stress tolerance in plants. Recently, maize HSBPs have been shown to influence raffinose content (Gu et al., 2019). Studies also have shown that raffinose accumulation is associated with drought tolerance in maize and Arabidopsis. Recently, Li et al. (2020) have shown that abiotic stresses, such as drought, salt, heat, and cold stresses, enhance the expression of a key enzyme (RAFFINOSE SYNTHASE, *ZmRAFS*) in raffinose biosynthesis, suggesting that HSBPs can regulate multiple stress pathways indirectly by regulating raffinose biosynthesis. In another study, ZmHSBP2 has been shown to interact with ZmHSFA2, a positive regulator of raffinose biosynthesis, leading to decreased accumulation of raffinose and eventually resulting in heat stress-sensitive phenotypes. Conversely, overexpression of *ZmHSFA2* in Arabidopsis has been found to enhance heat tolerance by improving raffinose biosynthesis (Gu et al., 2019). In Arabidopsis, *Athsbp1-KO* mutants exhibit acquired thermotolerance; however, *HSBP1* is also important for seed development, suggesting multiple roles for HSBPs in plants, although the mode of action remains unclear (Hsu et al., 2010). In maize, EMPTY PERICARP2 (*EMP2*), encoding HSBP1, has been found to be crucial for kernel development and embryogenesis (Fu et al., 2002). Moreover, *EMP2* and its paralog *HSBP2* have also been shown to exhibit differential regulation in maize development and heat stress response (Fu and Scanlon, 2004). The maize HSBP paralogs have also been found to exhibit different target specificity during interaction with maize HSFs, suggesting the possibility that they have non-redundant functions (Fu et al., 2006). It is likely that the roles of HSBPs may differ among paralogs and species, as their target specificity is not constant.

Chinese cabbage (*Brassica rapa* L. spp. *pekinensis*) is an economically important green leafy vegetable crop. It is a rich source of vitamins C and K, as well as several other nutrients, and is mainly cultivated in China, Korea, and Japan (Pang et al., 2018). Chinese cabbage production is constantly threatened by abiotic and biotic stress conditions, and these stress conditions are expected to worsen due to the climate crisis. Additionally, it is essential to note that seeds of Chinese cabbage plants are important genetic resources and are crucial for propagation, breeding, and germplasm maintenance. Seed development is a complex biological process, highly programmed by genetic constituents, and has been a major area of scientific research. Given the evidence in the literature supporting the involvement of HSBP genes in abiotic stress responses, including heat stress and seed development, we attempted to examine the biological significance of *HSBP* genes of *B. rapa* spp. *pekinensis* in this study. The *Brassica rapa* genome has undergone whole-genome triplication (WGT) or triploidization (Wang et al., 2011) during evolution. The events subsequent to WGT, such as gene fractionation, duplication (segmental and tandem), and transpositions, contribute to the contraction or expansion of a gene family with new genes and novel functionalities (Muthusamy et al., 2022). WGT has added two copies of *BrHSBP* in *B. rapa*, and their biological significance in plant development and stress responses is yet to be established.

Here, we present detailed functional analyses of *BrHSBP1* through transgenic overexpression (BrHSBP1^OX^) and the development of mutant lines (*Brhsbp1_KO*) using CRISPR-Cas9- mediated gene knockout approaches. Comparative analyses of transcriptomic signatures of control, BrHSBP1^OX^, and *Brhsbp1_KO* lines revealed that BrHBSP1 is primarily involved in seed and pod development via modulation of the plant’s reproductive structure development (RSD) genes. Additionally, yeast two-hybrid assay-based BrHSBP1-protein interaction studies revealed that BrHBSP1 may directly bind and interact with RSD and heat stress response genes.

## 2 Materials and methods

### 2.1 Gene identification and analysis of structural and molecular evolutionary characteristics

The Arabidopsis *HSBP1* (AT4G15802.1) sequence was used as query for the homology-based identification of *HSBP*s in the *Brassica rapa* genome (Brara Chiffu V 1.5 cds or Brara Chiifu V 3.5 cds), downloaded from http://brassicadb.agridata.cn/brad/. To achieve this, BLASTP was performed using the NCBI standalone BLAST tool, with the following command: ‘blastp -query query.fa -db db -outout.txt -outfmt “6 qseqid qlen sseqid salltitles pident mismatch gapopen qstart qend qcovs sstart send evalue bitscore” -evalue 0.00001 -max_target_seqs 20-num_threads 4’. BLAST hits were evaluated for the HSBP1 characteristic conserved domain (pfam06825) using the CD-search tool (Marchler-Bauer and Bryant, 2004). BLAST hits with the required CD in the same order as that of the queries were selected for further study. The gene structure and the “simple Ka/Ks Calculator (NG)” tool in Tbtools (Chen et al., 2020) was utilized to infer the presence of molecular evolutionary changes in BrHSBPs. Following Koch et al. (2000), the divergence time (in MYA, million years ago) of duplicated genes was calculated using the formula T = Ks/2r, where r is 1.5 × 10^−8^ synonymous substitutions per site per year, representing the rate of divergence for nuclear genes from plants.

### 2.2 Plant materials and stress treatments

The plant materials and treatment conditions used in this study were the same as those we have reported on previously (Muthusamy et al., 2021). Briefly, eight-day-old *B. rapa* (‘DH03’) seedlings (*n* = 5) grown in a hydroponic system were supplemented with phytohormones (each at 100 μM concentration), such as abscisic acid (ABA), indole-3-acetic acid (IAA), ethephon (ethylene), kinetin, gibberellic acid (GA_3_), salicylic acid (SA), and methyl jasmonate (JA); with hydrogen peroxide (10 mM H_2_O_2_); and with D-mannitol (350 mM). These seedlings were grown in a growth chamber for three consecutive days. Similarly, high- and low-temperature stresses were imposed by incubating seedlings in growth chambers at 37°C for 6 h and at 4°C for 6 h, respectively. Seedlings grown in Murashige and Skoog (MS) solution were used as controls. Additionally, the splicing patterns of *BrHSBP1* and *BrHSBP1-like* were profiled during exposure to high- and low-temperature stresses in two different growth periods (at 11 and 23 days old). All the seedlings that underwent these treatments were harvested, frozen in liquid nitrogen, and stored at -80°C for *BrHSBP1* and *BrHSBP1-like* gene expression analyses.

### 2.3 Expression profiling of *BrHSBP*s across stresses and in different tissues

Two micrograms (μg) of total RNA were extracted from each sample using the RNeasy Plant Mini Kit (QIAGEN, Germany). cDNA was prepared in 20 μl reactions with amfiRivert cDNA Synthesis Platinum Master mix according to the manufacturer’s instructions (GenDEPOT, Baker, TX, USA). For *BrHSBP* expression profiling, qRT-PCR was performed using the CFX96TM Real-Time PCR Detection System (Bio-Rad, California, USA) with primers specific to *BrHSBP1* (Bra038064) (FP-TGACATGGGAGGCAGAATCA; RP-GATTTGGAGGCAGCCGGA) and *BrHSBP1-like* (Bra012763) (FP-AGCAGATGCAAAGCAGGTTTC; RP-GATTTGGAGGCAGGAGTTGGA), along with AccuPower®2X GreenStar Master Mix (Bioneer, Daejeon, Korea). The qPCR conditions were as follows: 95°C for 5 min followed by 40 cycles of 95°C for 15 s and 58°C for 20 s. *BrActin2* (FP-CTCAGTCCAAAAGAGGTATTCT; RP-GTAGAATGTGTGATGCCAGATC) was used as an internal control. For tissue-specific expression analyses, cDNAs derived from the following tissue samples were used: root (primary (PR) and secondary (SR) tissues), leaf peduncle (LP), shoot (ST), leaves (young (YL) and old (OL) tissues), seed (SD), green silique (SQ), flower bud (FB), and flower (FL).

### 2.4 Construct development

The full-length coding sequence of *BrHSBP1* was amplified by polymerase chain reaction (PCR) with gene-specific forward (5′- AAAAAGCAGGCTATGGATGGGCATGATTCTGA-3′) and reverse (5′- AGAAAGCTGGGTCTAACTAGCCGGTGTTTTGGG -3′) primers. The PCR amplicons were initially digested with *SpeI* restriction enzymes, and the purified products (~0.3kb) were then infused with mGFP at the N-terminal region in the transgene orientation to the pCAMBIA1302 vector (pCAMBIA1390::35S-Pro+ BrHSBP1: mGFP) between the cauliflower mosaic virus 35S promoter (CaMV35Sp) and the nopaline synthase terminator site. The Gateway™ destination vector, pHAtC binary vector (Kim et al., 2016), was used to create *BrHSBP1* knock-out (*Brhsbp1-KO*) lines in *B. rapa*. Briefly, two guide RNAs were designed using the CRISPR-RGEN Tools package online (http://rgenome.ibs.re.kr) (Park et al., 2015), and the sgRNA templates were annealed using two sets of primers. sgRNA1 templates were annealed using GATTGAGAGCAGAGAT GGGAGTAGA (gRNA1_F): and AAACTCTACTCCCATCTCTGCTCTC (gRNA1_R) primers. Similarly, GATTGTCATCGCCTGATTTGGAGGG and AAACCCCTCCAAATCAGGCGATGAC were used for sgRNA2 templates. The sgRNAs were cloned into pHAtC using the *AarI* restriction enzyme. For expression, CaMV 35S was used for SpCas9, and U6/U3 for sgRNAs. The resultant binary vectors for overexpression (BrHSBP1^OX^) and knock-out of BrHSBP1 (*Brhsbp1_KO*) expression cassettes were genetically transformed into *B. rapa* cv. Dongbu (DB) using *Agrobacterium tumefaciens* (strain GV3101)-mediated T-DNA transformation methods.

### 2.5 Confocal microscopy

Abaxial epidermis peel tissues from the young leaves of three-week-old BrHSBP1^OX^ lines grown under greenhouse conditions were prepared for GFP visualization using a confocal laser scanning microscope (CLSM) (Leica SP8, Leica Microsystems). The Argon laser at 488 nm was used to excite the GFP and chlorophylls simultaneously, with the detection of emission spectra set to 495–520 nm and 630–700 nm for GFP and chlorophyll autofluorescence visualization (40× magnification), respectively. A nuclear-specific stain known as DAPI (4’,6-diamidino-2-phenylindole) was used at a final concentration of 1µg/ml for staining. Staining was carried out on abaxial leaf epidermis cells for 15 minutes; these were then washed twice with 1X PBS before being visualized with the DAPI filter set to use a 405 nm laser in the CLSM. The emission detection range was narrowed to 562–600 nm wavelength.

### 2.6 Screening and selection of BrHSBP1^OX^ and *Brhsbp1_KO* lines

Young leaves (~2cm) from potential *Brhsbp1_KO* (T0) mutants and controls growing in MSA medium were collected for extraction of genomic DNA using the DNeasy Plant Mini Kit (QIAGEN, Hilden, Germany). Using DNA as a template for each sample, *BrHSBP1* was amplified using primers (>pLSI84-85_F TGTGTGTTTCA GCAAACCAGG; >pLSI84-85_R GTAA TGTACCACCACA TCATCAAAT). Additionally, primers specific to Cas9 were used for confirmation of transgene integration into the host genome. The amplicons of potential mutant populations were sequenced using the Sanger sequencing method. The sequences of test samples were compared by aligning the sequences of wild-type lines to decipher the number of bases and the base quality of insertion/deletion mutants using the ICE Analysis tool by SYNTHEGO at https://ice.synthego.com/#/. For phenotyping, the seeds of selected BrHSBP1^OX^ and non-transgenic control lines were surface-sterilized and plated on half-strength MS Agar (MSA) medium in triplicate. After one-day stratification at 4°C under dark conditions, the seeds on MSA plates were incubated in a controlled plant tissue culture room (16 h light at 25°C and 8 h dark at 23°C; light intensity: 100 to 120 μmol m^−2^ s^−1^) for germination. After 5 days post-germination, the seedlings were transferred to greenhouse conditions. Leaf samples from 3-week-old *B. rapa* ‘DH03’ (DB) seedlings (non-transgenic controls) (n=5) and BrHSBP1^OX^ lines maintained in greenhouse conditions were utilized for analyses of *BrHSBP1* and its potential downstream gene expression. At maturity, the lengths of the seeds and pods of BrHSBP1^OX^ and non-transgenic controls (n=20) were measured. Meanwhile, DH03 seedlings (in triplicate) were transferred to multiwall plastic trays containing soil mixure and kept under greenhouse conditions for drought stress phenotyping studies. After plant establishment (1 week), progressive drought stress was imposed by withholding of irrigation for 17 consecutive days; the green phenotypes were noted before and after drought recovery through watering for one day. The drought-stressed BrHSBP1^OX^ was used for analyses of raffinose biosynthesis pathway gene expression. Primer sequences are listed in **Supplementary Table S1**.

### 2.7 RNA-seq, Gene Ontology, functional annotation, and statistical enrichment analyses

Total RNA was extracted from 100 mg powdered tissues (leaves) of *BrHSBP1^OX^*, *Brhsbp-KO*, and wild-type (control, CT) lines (*n* = 3) using the RNeasy Plant Mini Kit (QIAGEN, Hilden, Germany) according to the manufacturer’s instructions. Total RNA quantity and quality was assessed using a NanoDrop 2000 spectrophotometer (Thermo Scientific Co., Waltham, MA, USA) and Bioanalyzer 2100 (Agilent Technologies, Santa Clara, CA, USA). Five micrograms of each RNA sample was used to generate nine cDNA libraries (using the TrueSeq Stranded mRNA Prep Kit) containing inserts that were approximately 150–200 bp in size. For RNA sequencing, 101-nucleotide paired-end sequencing (*n* = 3) was conducted using an Illumina NovaSeq6000 platform (Illumina, San Diego, CA, USA) by Phyzen (Seongnam, Korea). Raw RNA reads (~4.6GB or more for each sample) were filtered and trimmed using the Trimmomatic v0.39 Toolkit (Bolger et al., 2014) to remove low-quality bases (>30) and adapter sequences. An additional decontamination process was carried out using the BBDuk program (https://jgi.doe.gov/data-and-tools/bbtools/bb-tools-userguide/bbduk). Preprocessed reads were mapped to Brara_Chiifu_V3.5.fa and the annotated gene model v.3.5 (available at https://www.Brassicadb.cn/) using HISAT2 (v2.1.0) (Kim et al., 2019) with default parameters. The number of mapped reads in the gene regions was counted using the HTSeq-count method to profile the expression value of each gene. Raw read counts were normalized, and differential gene expression analysis was performed using DESeq2 (Love et al., 2014). Differentially expressed genes (DEGs) with FDR < 0.05 and fold change (logFC) ≥ |2| were considered significant and used for further analyses (**Supplementary Data Sheet 1**). Functional annotation was carried out with the assistance of DIAMAND BLAST (Buchfink et al., 2014) using the NCBInr, SWISSPROT, BLAST2GO (Conesa et al., 2005), and InterProScan (Quevillon et al., 2005) databases. Gene ontology (GO) terms and functional enrichment of DEGs were obtained using the gProfiler web tool (Raudvere et al., 2019). Overrepresentation and GO term enrichment analyses were performed under three major categories (biological processes, molecular biology components, and cellular components) for different comparisons, including CT vs. BrHSBP1^OX^, CT vs. *Brhsbp1-KO*, and BrHSBP1^OX^ vs. *Brhsbp1-KO* libraries (**Supplementary Data Sheet 2**).

### 2.8 Yeast two-hybrid assays

For yeast two-hybrid (Y2H) assays, the full-length coding sequences of *BrHSBP1*, *BrHSBP1-like*, flowering/reproductive structure development-associated candidate genes (*bHLH* (BraA05g013660), *SPL8* (BraA09g068100), *SEP3* (BraA07g012730), *ARPN* (BraA02g037040), *TPD1* (BraA01g015750), and *AGL15* (BraA09g026780)), HSR pathway genes (*BrHSFA1e1*, *BrHSFA1d1*, *BrHSFA1b2*, *BrHSFA1a*, *BrHSFA1d2*), and the raffinose biosynthesis pathway gene (*BrGolS7*) were amplified using PrimeSTAR^®^ Max premix (Takara, Japan) and gene-specific primer sets (**Supplementary Table S2**). Both *BrHSBP1* and *BrHSBP1-like* were cloned in fusion with both GAL4-AD and GAL4-BD, and all other genes examined were cloned in fusion with GAL4-AD only. All plasmid combinations were initially transformed into DH5 alpha *E. coli* cells to facilitate plasmid amplification and transgene verification via Sanger sequencing. The resultant prey and bait vectors were transformed into MaV203 yeast cells according to the instructions provided by Clontech. Double transformants expressing BrHSBP1 or BrHSBP1-like and candidate interacting proteins were grown on synthetic dropout (SD) growth media without leucine and tryptophan (SD/-Leu/-Trp) and further screened on SD/-Leu/-Trp/-His/-Adenine medium (SD medium without leucine, tryptophan, histidine, and adenine) supplemented with 15 mM 3-amino-1,2,4-triazole (3-AT) (SD/-Leu/-Trp/-His/-Adenine/+ 3-AT) to verify their interaction. The concentration of 3-AT was optimized to suppress the self-activation of reporter genes in double transformants containing BrHSBP1-GAL4-BD and empty GAL4-AD vectors. Yeast cells transformed with pGBKT7-P53/pGADT7-T (P53/T) (interactions between p53 and the SV40 large T-antigen) were used as positive controls, and cells transformed with pGBKT7-Lam/pGADT7-T (Lam/T) (lamin and the SV40 large T-antigen) were used as negative controls.

## 3 Results

### 3.1 Structural and evolutionary characteristics of BrHSBPs

The homology-based gene identification approach with *AtHSBP1* showed two segmental duplicates, which were designated as *BrHSBP1* and *BrHSBP1-like*, present in the *B. rapa* genome (Brara Chiffu V 1.5 cds, available at http://www.brassicadb.cn/#/BLAST/). Comparison of the peptide sequences of BrHSBP1 and BrHSBP1-like with AtHSBP1 (AT4G15802.1) revealed that those sequences had high degrees of similarity of 100% and 95%, respectively, with AA variations positioned at 9, 10, 73, 85, and 87. Interestingly, the characteristic HSBP domains of BrHSBPs were found to have 99–100% similarity to their orthologs. *BrHSBP1* and *BrHSBP1-like* were found on different chromosomes (A8 and A3). The nucleotide sequences of both *BrHSBP1* and *BrHSBP1-like* isolated from *B. rapa* (‘DH03’) in this study were submitted to the NCBI gene database under accession numbers MT514663.1 (similar to Bra038064/BraA08g011940.3.5C) and MT514662.1 (similar to Bra012763/BraA03g047020.3.5C), respectively. Comparison of *BrHSBP* gene structures (e.g., number of exons, their order and their types (symmetrical and asymmetrical)) with *AtHSBP1* revealed that they have undergone evolutionary structural changes and gene diversification after divergence (**Supplementary Figure S1**). *BrHSBP1* has two symmetrical exons (0,0 class). In comparison, *BrHSBP1-like* has four exons, consisting of a pair of symmetrical exons (0,0 class) and a pair of asymmetrical exons (0,1 class), whereas *AtHSBP1* has five exons (0,0,0,0,2 class).

### 3.2 Relative quantification of *BrHSBPs* in different tissues and during different treatments

**Figure 1:**
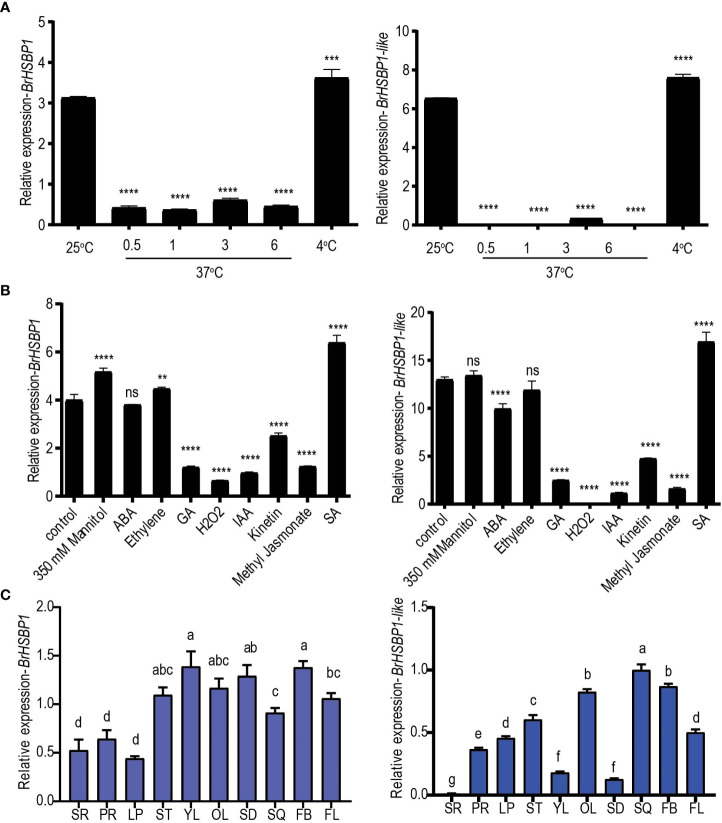
Expression profiling of *BrHSBP1* and *BrHSBP1-like* across different abiotic stresses, exogenous phytohormones, and tissues. **(A)** Changes in expression of *BrHSBP1* and *BrHSBP1-like* under different temperature regimes (25°C, 37°C at different time points of exposure (0.5, 1, 3, and 6 hr), and 4°C). **(B)** Changes in expression of *BrHSBPs* during exogenous application of phytohormones (ABA, ethylene (ethophen), gibberellin, IAA, kinetin, methyl jasmonate, and salicylic acid), 350 mM mannitol, and hydrogen peroxide to eight-day-old seedlings growing under optimal growth conditions. **(C)** Relative quantification of *BrHSBPs* in different tissue samples, including the root (primary (PR) and secondary root (SR) tissues), leaf peduncle (LP), shoot (ST), leaves (young (YL) and old leaf (OL) tissues), seeds (SD), green siliques (SQ), flower buds (FB), and flowers (FL). Significant differences (indicated by a, b, and c) were assessed via one-way ANOVA. *p < 0.05; **p < 0.01; ***p < 0.001; ****p < 0.0001. ns, non-significant.

In response to high-temperature stress (37°C) at different time intervals, transcript levels of both *BrHSBP1* and *BrHSBP1-like* were significantly reduced compared to the levels in controls grown at 25°C (**Figure 1A**). However, exposure to cold conditions (4°C) upregulated *BrHSBP1* and *BrHSBP1-like*. Exposure to GA_3_, H_2_O_2_, IAA, kinetin, and JA downregulated both genes (**Figure 1B**), while exposure to SA significantly upregulated them. In contrast, the stress hormone ABA upregulated the expression of *BrHSBP1* but not *BrHSBP1-like*, while exposure to 350 mM mannitol and ethylene downregulated *BrHSBP1*-*like* only. Moreover, spatiotemporal patterns of expression among different tissues of *B. rapa* indicated that *BrHSBP1* was most predominant in flower buds and young leaves, followed by seeds (**Figure 1C**), whereas *BrHSBP1-like* was most predominant in green siliques, followed by flower buds. Unlike *BrHSBP1*, the relative expression of *BrHSBP1-like* was minimal in seeds and young leaves, possibly suggesting a differential role in development. Additionally, the lowest level of expression of *BrHSBP1* was found in the leaf peduncle, while *BrHSBP1-like* was least expressed in secondary roots. The splicing patterns of *BrHSBP1* and *BrHSBP1-like* under exposure to high- and low-temperature conditions were also profiled in 11-day-old whole *B. rapa* seedlings exposed to different temperatures for different periods of time (**Supplementary Figure S2**). The result showed that heat stress (37°C) altered the splicing of *BrHSBP*s irrespective of the duration of exposure, while the low-temperature conditions (4°C) did not alter the splicing status of *BrHSBP*s. However, when 21-day-old leaf samples were exposed to similar conditions, they revealed different splicing patterns for each gene, indicating that alternative splicing of *BrHSBP*s under exposure to heat stress may also depend on the developmental stage of the plant (**Supplementary Figure S2**). The BrHSBP1 and BrHSBP1-like splice variants had sequence coverages of 45% and 26%, respectively, with BrHSBP1 and BrHSBP1-like genes. Although their roles are yet to be established, there is a high possibility that BrHSBP1, with its incomplete domain sequences (lost 74 nt), undergoes the nonsense-mediated mRNA decay pathway. Interestingly, BrHSBP1-like splice variants had a high sequence coverage of 84% with an uncharacterized ncRNA (LOC117133031) of *B. rapa*. The regulatory function or biological significance of LOC117133031 is yet known. BrHSBP1-like variants did not possess characteristic HSBP1domains as expected in ncRNA.

### 3.3 Development and seed phenotyping of BrHSBP1^OX^ and *Brhsbp1_KO B. rapa* lines

**Figure 2:**
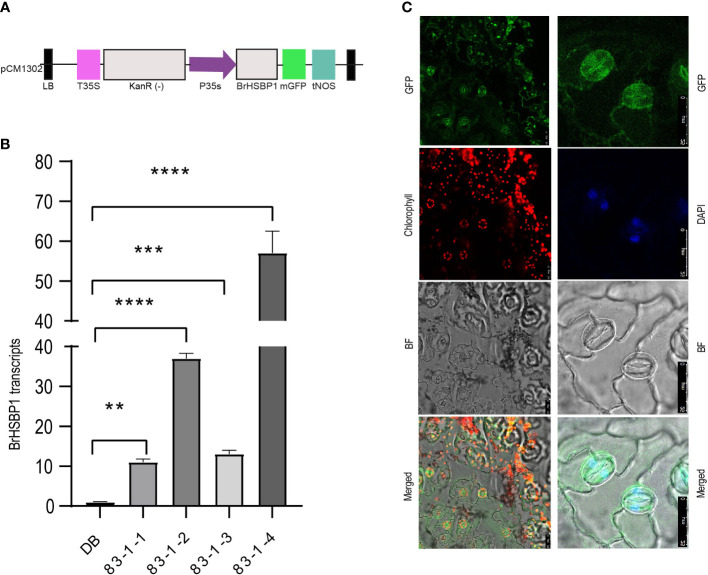
Expression activity of the GFP-tagged BrHSBP1 signal was localized to the nuclei and cytosol. **(A)** A schematic representation of the expression cassette designed for overexpression of BrHSBP1. **(B)** Relative expression levels of BrHSBP1 in BrHSBP1^OX^ lines. **(C)** Confocal microscopic images showing the GFP-tagged BrHSBP1 signal in abaxial epidermis peels of BrHSBP1^OX^ lines. i) The different panels labeled GFP, chlorophyll, and bright field represent the corresponding signals for the same samples. ii) Magnified stomata show the GFP-BrHSBP1 signals in nuclei and cytosol; this was confirmed by nuclei-specific DAPI (4’,6-diamidino-2-phenylindole) staining at a final concentration of 1µg/ml. Scale bar = 25 µm. *p < 0.05; **p < 0.01; ***p < 0.001; ****p < 0.0001.

**Figure 3:**
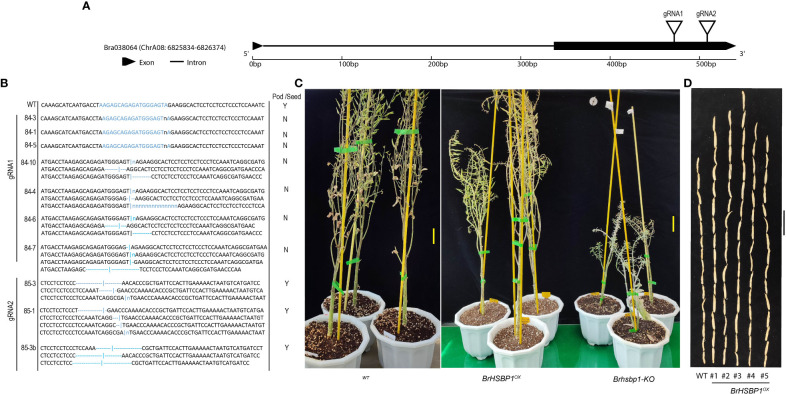
Seed and pod phenotypes of BrHSBP1^OX^ and *Brhsbp-KO* lines of *Brassica rapa* sp. *pekinensis*. **(A)** *BrHSBP1* gene structure denoting the gRNA target positions. **(B)** Some of the representative BrHSBP1-KO mutant genotypes. **(C)** Representative images of matured wild-type (WT), BrHSBP1^OX^, and *Brhsbp-KO* lines. **(D)** The seeds (20 in number) of WT and BrHSBP1^OX^ lines. Scale bars = 5 cm.

**Figure 4:**
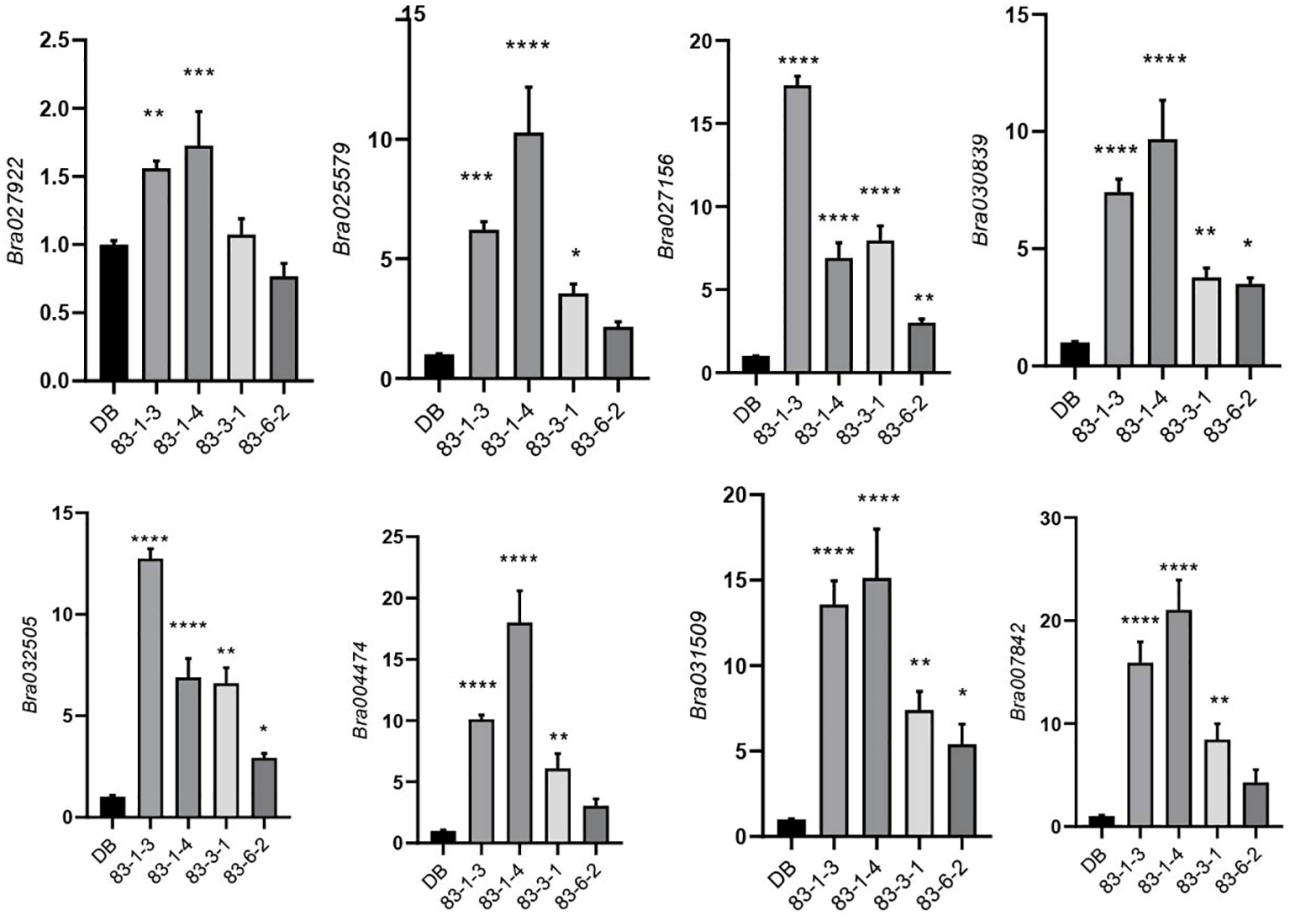
Expression profiling of raffinose biosynthesis pathway genes in BrHSBP1^OX^ lines, showing relative expression levels of raffinose biosynthesis pathway genes Bra004474 [*GolS1*], Bra007842 [*GolS6*], Bra025579 [*RS5*], Bra027156 [*GolS4*], Bra027922 [*GolS2*], Bra030839 [*RS1*], Bra031509 [*GolS7*], Bra031663 [*GolS3*], and Bra032505 [*GolS5*] genes in wild (DB) and BrHSBP1^OX^ lines (83-1-3,4,83-3-1, and 83-6-2). *BrACT* was used for normalization of gene expression levels. The relative expression profile was derived from qRT-PCR of three independent wild-type lines and *BrHSBP1^OX^* lines. Expression was normalized to the Actin gene. Asterisks represent statistical significance as assessed via one-way ANOVA: *p < 0.05; **p < 0.01; ***p < 0.001; ****p < 0.0001.

We selected a total of four T_3_ BrHSBP1^OX^ lines through screening by qRT-PCR and a total of 23 T_0_ *Brhsbp1_KO* lines through targeted gene sequencing. *BrHSBP1* overexpression ranged from 10- to 50-fold (**Figure 2A**) in transgenic lines that were selected for phenotyping and molecular biology studies. The mutant population yielded seven homozygous *Brhsbp1_KO* lines, while 16 lines were heterozygous mutants. The vegetative growth phase of BrHSBP1^OX^ lines did not show morphological differences with CT; however, distinguishable seed phenotypes at maturity were observed. Almost all of the selected T_3_ BrHSBP1^OX^ lines exhibited enlarged seeds or pods (**Figure 3**; **Supplementary Figure S3**) compared to CT. Interestingly, none of the T_0_ homozygous mutants developed in this study was able to produce seeds or pods; all were completely aborted (**Figure 3**). In comparison, heterozygous mutants produced very few seeds compared to CT. Targeted gene sequencing of *Brhsbp1_KO* lines indicated that any InDels around the first gRNA region (AAGAGCAGAGATGGGAGTA) in the *BrHSBP1* resulted in complete seed abortion (**Table 1**). Additionally, drought stress phenotyping of T_3_ BrHSBP1^OX^ lines revealed that the green phenotype was relatively widely retained under BrHSBP1 overexpression, thus indicating improved stress tolerance in transgenic lines (**Supplementary Figure S4**). Gene expression analyses of drought-stressed BrHSBP1^OX^ showed that almost all of the raffinose biosynthesis pathway-related genes under study were upregulated compared to controls (**Figure 4**).

**Table 1:** List of floral organ genes downregulated in non-transgenic controls vs. *brhsbp1-KO*.

Gene ID	Gene Name	Log2FC	p-value	p_adj_
BraA09g026780.3.5C	AGAMOUS-like 15 (AGL15)	-3.2	0.0076	0.0393
BraA05g005280.3.5C	AGAMOUS-like 6 (AGL6)	-8.0	0.0000	0.0001
BraA03g024420.3.5C	AP2/B3-like transcriptional factor family protein (NGA1)	-2.1	0.0022	0.0153
BraA04g027390.3.5C	ATP binding cassette subfamily B1 (ABCB1)	-2.4	0.0000	0.0000
BraA03g061190.3.5C	Cytochrome P450 superfamily protein (ROT3)	-2.3	0.0050	0.0289
BraA10g030030.3.5C	ERECTA-like 2 (ERL2)	-2.0	0.0008	0.0069
BraA07g012730.3.5C	K-box region and MADS-box transcription factor family protein (SEP3)	-3.6	0.0000	0.0000
BraA09g025050.3.5C	K-box region and MADS-box transcription factor family protein (SEP4)	-3.0	0.0000	0.0000
BraA05g013580.3.5C	LIGHT-DEPENDENT SHORT HYPOCOTYLS-like protein (DUF640) (LSH3)	-2.0	0.0006	0.0054
BraA01g009640.3.5C	Leucine-rich repeat protein kinase family protein (SUB)	-2.4	0.0000	0.0000
BraA05g041190.3.5C	Alpha/beta-hydrolases superfamily protein (GID1A)	-3.0	0.0004	0.0040
BraA05g013660.3.5C	Basic helix-loop-helix (bHLH) DNA-binding superfamily protein (AT2G31220)	-3.7	0.0000	0.0006
BraA03g018810.3.5C	Cell wall invertase 4 (cwINV4)	-2.3	0.0053	0.0302
BraA06g009950.3.5C	Cytochrome P450, family 78, subfamily A, polypeptide 5 (CYP78A5)	-4.6	0.0000	0.0000
BraA06g009360.3.5C	Hypothetical protein (AT1G12380)	-2.5	0.0000	0.0001
BraA02g037040.3.5C	Plantacyanin (ARPN)	-2.3	0.0042	0.0252
BraA09g068100.3.5C	Squamosa promoter binding protein-like 8 (SPL8)	-4.3	0.0018	0.0131
BraA04g030840.3.5C	Squamosa promoter binding protein-like 9 (SPL9)	-3.1	0.0000	0.0000
BraA01g015750.3.5C	Tapetum determinant 1 (TPD1)	-2.7	0.0000	0.0000
BraA09g060980.3.5C	Xyloglucan endotransglucosylase/hydrolase 28 (XTH28)	-2.4	0.0000	0.0002

### 3.4 Cellular localization of BrHSBP1-GFP

The cellular localization of the BrHSBP1-GFP protein in the leaf abaxial epidermis cells of stable transgenic *B. rapa* lines was investigated via confocal laser scanning microscopy (**Figure 2B**). Microscopic analyses of guard cells revealed that BrHSBP1-GFP is likely located in the nuclei and cytosol. To confirm the presence of BrHSBP1-GFP proteins in the nuclei of guard cells, DAPI (4’,6-diamidino-2-phenylindole) was used as a nuclear-specific stain, at a final concentration of 1µg/ml. Overlapping of DAPI-stained nuclei in the DAPI panel confirmed that most of the GFP signals in GFP panels represent the nuclei/DNA of different cell types, thus clearly indicating that BrHSBP1 is localized to the nucleus and destined to function in the nucleus.

### 3.5 Transcriptomic signatures of wild, BrHSBP1^OX^, and *Brhsbp1_KO* lines

**Figure 5:**
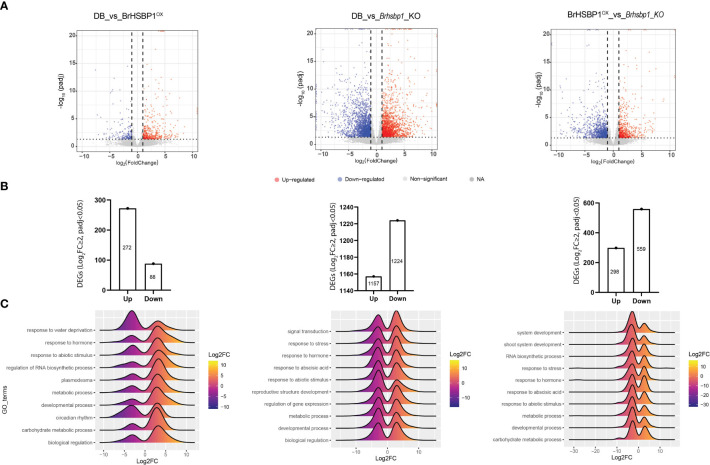
The transcriptomic signatures of BrHSBP1^OX^ and *Brhsbp1-KO* reveal differential gene expression of plant reproductive structure developmental genes. **(A)** Volcano plots representing the differentially expressed genes in comparisons between transcriptomes of DB vs. BrHSBP1^OX^, DB vs. Brhsbp1_KO, and rHSBP1^OX^ vs. Brhsbp1_KO. **(B)** Bar charts indicating the number of significantly differentially expressed genes (Log_2_FC≥2 and p_adj_<0.05) in the indicated comparisons. **(C)** Ridgeline plots depicting the differential regulation of key biological processes identified during comparative transcriptomic analyses of DB, BrHSBP1^OX^, and *Brhsbp1_KO* libraries.

To understand the transcriptomic dynamics that may be responsible for enlarged seed in BrHSBP1^OX^ and the no-seed phenotypes seen in *Brhsbp1-KO* lines, RNA-seq based transcriptomic data from these lines were compared with that of the CT (**Figure 5**). A total of 113.28, 109.47, and 112.82 million clean/filtered reads were produced from control, BrHSBP1^OX^, and *Brhsbp1-KO* libraries, respectively. These reads were mapped onto *B. rapa* reference genome sequences and assembled into 47,249 transcriptional units (TUs), which accounted for 34,133 non-redundant genes. Differential gene expression analyses using DESeq2 revealed that the expression of 671 and 4727 TUs was significantly altered (log_2_FC ≥ 1; *p* < 0.05) in the BrHSBP1^OX^ and *Brhsbp1-KO* libraries, respectively, compared to the control library (**Figure 4A**). A similar comparison between BrHSBP1^OX^ and *Brhsbp1-KO* libraries indicated that 1,779 TUs were significantly altered. The raw sequence reads were made available in the NCBI Sequence Read Archive (SRA) under BioProject accession number PRJNA905169. Statistical enrichment analysis of 307, 1954, and 718 differentially expressed genes (DEGs) (log_2_FC ≥ 2; FDR < 0.05) with known Gene Ontology (GO) terms from comparisons of CT vs. BrHSBP1^OX^, CT vs. *Brhsbp1_KO*, and BrHSBP1^OX^ vs. *Brhsbp1_KO* (**Figure 4B**) revealed that transcripts associated with several molecular biology components, multiple biological processes, and cellular components were enriched. In particular, DEGs related to water deprivation response and circadian rhythms were highly reduced in BrHSBP1^OX^ (**Figure 4C**), while TUs associated with hormone response, abiotic stimulus response, regulation of RNA biosynthetic processes, metabolic and biological regulation, and developmental processes were enriched. In contrast, genes associated with multiple responses were highly reduced in *Brhsbp1_KO*, including developmental/reproductive structure development, with the exception of genes associated with signal transduction mechanisms. Finally, in the comparison between BrHSBP1^OX^ and *Brhsbp1_KO*, many of the key response-associated genes, such as shoot system development, RNA biosynthetic processes, stress response, hormone response, abiotic stress response, metabolic processes (esp. carbohydrate metabolic process), and developmental processes, were reduced in *Brhsbp1_KO*.

#### 3.5.1 Expression pattern of the reproductive structure development process

To gain deeper insight into the seedless phenotypes of *Brhsbp1_KO*, we conducted a detailed analysis of the expression patterns of genes associated with reproductive structure development (**Supplementary Data Sheet 3**). We identified a total of 133 DEGs known to be linked with reproductive structure development in *Brhsbp1_KO* lines. Of these, 81 were downregulated, while 52 were upregulated. Notably, some of the highly downregulated genes included pyridoxal phosphate (PLP)-dependent transferases (*POP2*), AGAMOUS-like 6 (*AGL6*), MLP-like protein 328 (*MLP328*), *AGL20*, mannose-binding lectin superfamily protein (*JR1*), UDP-glycosyltransferase (*SGT*), cold/circadian rhythm/RNA binding 2 (*GRP7*), *AGL19*, gibberellin 20-oxidase 3 (*GA20OX3*), and EARLY FLOWERING-like protein (*ELF4*). In contrast, the top upregulated genes comprised beta-xylosidase 1 (*BXL1*), RmlC-like cupins superfamily protein, thioredoxin superfamily protein (*ROXY2*), 1-amino-cyclopropane-1-carboxylate synthase 7 (*ACS7*), *ACS11*, actin-related protein 4 (*ARP4*), and K-box region and MADS-box transcription factor family protein (*AGL72*). Futhermore, we observed a reduction in the expression of specific flower development initiation genes in the comparison between BrHSBP1^OX^ and *Brhsbp1_KO*, including squamosa promoter binding protein-like 9, *AGL20*, and basic leucine zipper (bZIP) transcription factor family protein (*FD*). Conversely, two other flowering-related genes, *RAV2* and AP2/B3 transcription factor family protein (*TEM1*), were induced in the *Brhsbp1_KO* line.

#### 3.5.2 Expression pattern of shoot and other developmental processes

A total of 61, 314, and 132 developmental DEGs were identified in comparisons of CT vs. BrHSBP1^OX^, CT vs. *Brhsbp1_KO*, and BrHSBP1^OX^ vs. *Brhsbp1_KO*, respectively. Of these, 55.1–66.6% were downregulated in *Brhsbp1_KO* when compared to the CT or BrHSBP1^OX^ lines. Additionally, 48 DEGs (accounting for 36.3% of the total) were known to be involved in shoot development. Among these, expression of 72.9% was reduced in *Brhsbp1_KO* lines compared to BrHSBP1^OX^ lines. However, only 24.5% of the developmental genes were downregulated in BrHSBP1^OX^ compared to CT lines. To examine the biological significance of *BrHSBP1* in stress and hormonal responses, GO terms indicating a role in stress and hormones were analyzed for their expression pattern in transgenic lines over the controls. Interestingly, *BrHSBP1* overexpression did not significantly elicit guidelines, whereas *Brhsbp1_KO* altered the expression of 180–450 transcripts, as identified based on two different comparative analyses with CT or BrHSBP1^OX^ libraries. Of these, 51–62% exhibited a declining trend. In regard to hormone response DEGs, 41, 221, and 86 were reported in comparisons of CT vs. BrHSBP1^OX^, CT vs. *Brhsbp1_KO*, and BrHSBP1^OX^ vs. *Brhsbp1_KO*, respectively. A higher number of DEGs were upregulated in BrHSBP1^OX^, while a higher number of DEGs were downregulated in *Brhsbp1_KO*. Among GA response DEGs, the gibberellin-regulated family protein (*GASA6*) was upregulated in BrHSBP1^OX^. In contrast, six gibberellin genes were differentially expressed in *Brhsbp1_KO*. Of these, the expression of gibberellin oxidase family genes, namely *GA2ox2*, *GA2ox8*, *GA2ox6*, *GA3ox1*, and AT1G22690.1_gibberellin-regulated family protein (*GASA9*), were enhanced. Two other genes, *GA20ox3* and gibberellin 2-beta-dioxygenase (AT1G78440.1), were found to be reduced compared to CT. Moreover, expression data for the comparison of BrHSBP1^OX^ vs. *Brhsbp1_KO* revealed that *GA2ox8* was upregulated 5.39-fold, while *GA20ox3* was reduced 4.3-fold in expression.

### 3.6 Analysis of the interaction of BrHSBP1 with plant reproduction- or flower-associated candidate genes

**Figure 6:**
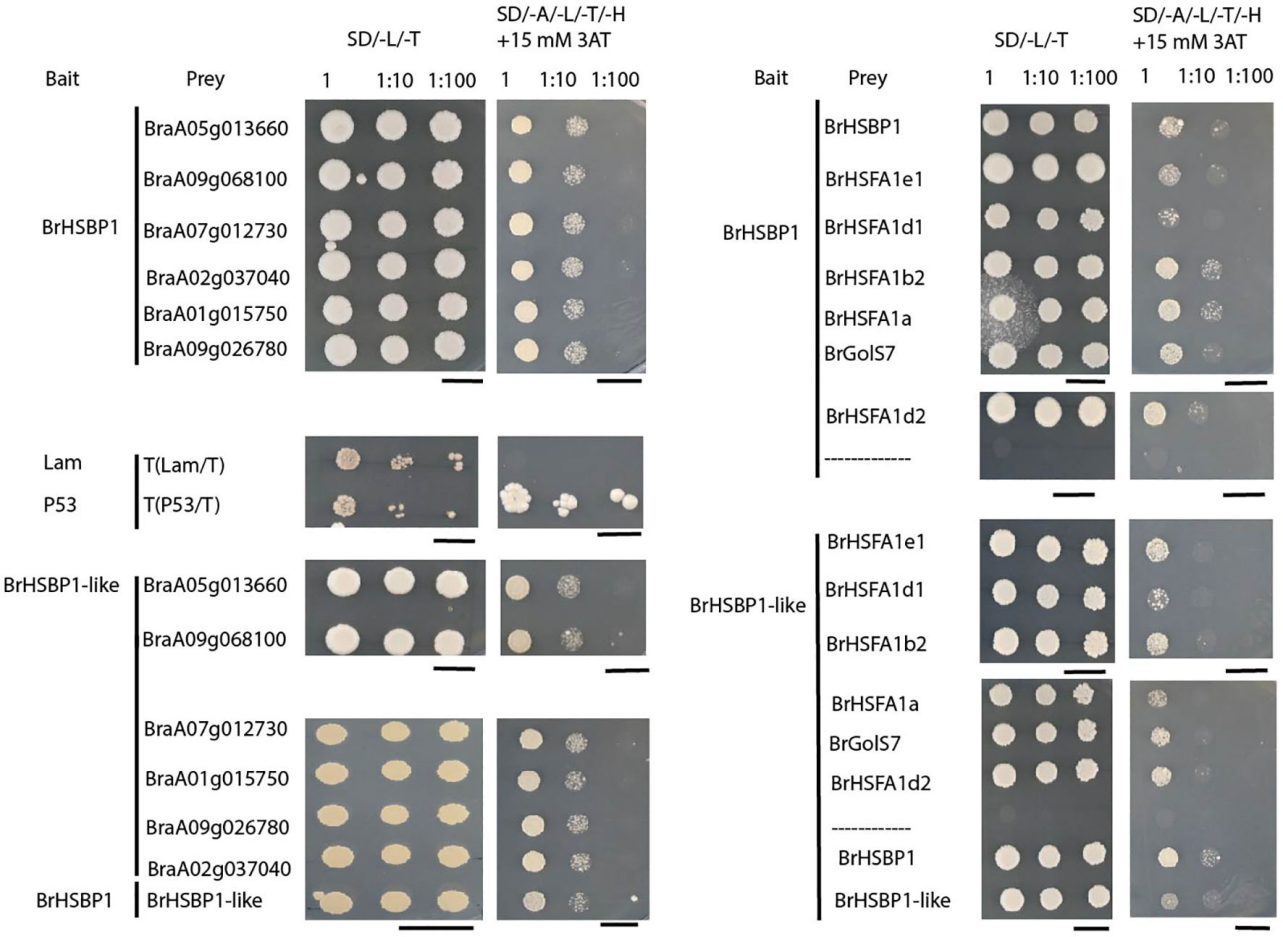
Protein–protein interactions of BrHSBPs with plant reproductive structure development-related genes and others. The figure represents yeast two-hybrid assays with cells harboring the indicated constructs grown on DDO (SD/-L/-T) or QDO supplemented with 15 mM 3-amino-1,2,4-triazole (3-AT) (SD/-A/-L/-T/-H+ 15mM 3AT) medium to verify their interaction. Yeast cells transformed with pGBKT7-P53/pGADT7-T (P53/T) (interactions between p53 and the SV40 large T-antigen) were used as positive controls, and cells transformed with pGBKT7-Lam/pGADT7-T (Lam/T) (lamin and the SV40 large T-antigen) were used as negative controls. Scale bars = 5 cm.

Downregulation of flowering/reproductive structural development-related genes in *Brhsbp1_KO* (Supplementary Data Sheet S3) and its seedless phenotypes prompted us to investigate the mode of regulation. BrHSBP1 can interact with target proteins through its central coiled-coil domain/HSBP1 (pfam06825) (Tai et al., 2002). Therefore, to determine whether BrHSBPs directly interact with flowering- or seed development-associated genes to modulate their expression, Y2H interaction tests were performed with six of the differentially expressed genes: BraA05g013660 (*bHLH010*), BraA09g068100.3.5C (*SPL8*), BraA07g012730.3.5C (*SEP3*), BraA01g015750.3.5C (*TPD1*), BraA09g026780.3.5C (*AGL15*), and BraA02g037040.3.5C (*ARPN*) (**Figure 6**). All of these proteins exhibited interactions with BrHSBP1 and BrHSBP1-like. Moreover, it was evident that BrHSBP1 can interact with itself as well as with BrHSBP1-like. However, BrHSBP1-like did not demonstrate self-interaction. Furthermore, to understand the regulation of HSR genes by BrHSBP1, we investigated the potential interactions of a small number of BrHSFs (HSFA1e1, HSFA1d1, HSFA1b2, HSFA1a, and HSFA1d2) with BrHSBP1/BrHSBP1-like proteins. The results indicated that BrHSBP1 interacted with HSFA1b2, HSFA1a, and HSFA1d2 proteins. Interactions with HSFA1e1 and HSFA1d1 were weak and negligible. A similar analysis with BrHSBP1-like proteins revealed interactions with HSFA1b2 and HSFA1d2, but not with HSFA1d1. Notably, BrHSBP1-like was found to interact with HSFA1e1, although its interaction with HSFA1a was weak. Beyond this, both BrHSBPs were found to interact with the raffinose biosynthesis protein BrGolS7, indicating their possible role in raffinose biosynthesis in *B. rapa* plants.

## 4 Discussion

In general, plant HSBP proteins have long been known for their association with heat stress response pathways. In particular, HSBP1 proteins bind with several heat shock proteins, including HSFA1a, a master regulator of heat stress response gene expression (Hsu et al., 2010). HSBP1 proteins negatively regulate these genes. Arabidopsis and maize HSBP mutations confer acquired heat stress tolerance phenotypes, thus reaffirming their negative regulation of HSR genes. However, other studies have indicated that HSBPs can also play a role in developmental processes (Fu et al., 2002; Fu and Scanlon, 2004; Fu et al., 2006). The role or biological significance of BrHSBPs in plant growth and development, or in abiotic stress responses, was not previously known. Therefore, this study was designed to reveal the biological significance of BrHSBPs in *B. rapa* plants through the combined application of transgenic technology and CRISPR-Cas gene editing methods. Preliminary studies showed that genome duplication and the subsequent fractionation events have added two *HSBP* genes in *B. rapa*. Based on their phylogenetic relationships with *AtHSBP1*, these were designated as *BrHSBP1* and *BrHSBP1-like* genes. The gene structure differences between *AtHSBP1* and *BrHSBPs*, or within *BrHSBPs*, suggest the possibility of evolutionary structural changes or gene diversification after the divergence. The presence of a symmetrical exon in *BrHSBP1* indicates possible exon shuffling, which could have resulted in only two exons. In preliminary studies involving exposure to heat stress (37°C) at different times during the vegetative growth phase, the expression of *BrHBSP1* and *BrHSBP1-like* was significantly reduced, in contrast with the expression pattern of *AtHSBP1* in Arabidopsis (Hsu et al., 2010). Cold treatment, on the other hand, enhanced the expression of both genes when compared to controls, indicating that *BrHSBPs* may be involved in temperature stress responses. Research on the biological significance of HSBP1 under conditions of abiotic stress has largely been limited to high-temperature stress, and further study is essential to unearth the role of these genes in other abiotic stress responses. Additionally, spatiotemporal expression profiling of *BrHBSPs* in different tissues showed that they were most abundant in floral buds, siliques, and young leaves, suggesting their possible contribution to plant growth and development in addition to abiotic stress responses. Rice HSBP homologs (*OsHSBP1* and *OsHSBP2*) are predominantly found in panicles (Rana et al., 2012), while ZmHSBP2 and EMP2 of maize show differential tissue preferences and responses to heat stress (Fu et al., 2006).

Furthermore, *BrHSBPs* showed differential expression in response to the exogenous application of phytohormones, particularly ABA and ethylene, and in response to 350 mM mannitol treatment, suggesting differential roles for the segmental duplicates observed in the *B. rapa* genome. The gene expression pattern of *BrHSBP1* in floral buds and young leaves, as well as the induced expression under exposure to 350 mM mannitol treatment, prompted us to study its role in more detail. For this purpose, we developed BrHSBP1^OX^ and *Brhsbp1*_*KO* lines using transgenic approaches and CRISPR-Cas9 editing tools. We also studied their cellular localization using GFP-tagged BrHSBP1 in stable transgenic BrHSBP1^OX^ lines, revealing that BrHSBP1 is destined to work in the nuclei and cytosol. Previous reports in the literature have indicated that elevated ZmHSBP2 in Arabidopsis negatively regulates raffinose biosynthesis (Gu et al., 2019). In this study, we found that most of the raffinose biosynthesis pathway genes under investigation were upregulated in BrHSBP1^OX^ lines under progressive drought conditions (**Figure 4**). However, none of the genes showed significant expression changes in large-scale comparative transcriptome studies of BrHSBP1^OX^ and CT under optimal conditions, possibly indicating that BrHSBP1 modulates raffinose biosynthesis-related genes in response to drought conditions. Before this study, HSBPs of Arabidopsis (Hsu et al., 2010), maize (Gu et al., 2019), tomato (Marko et al., 2019), and rice (Rana et al., 2012) had been studied for their role in heat stress response and in growth and development in plants. Overexpression of *AtHSBP1*, *ZmHSBP2*, *OsHSBP1*, and *OsHSBP2* have been found to contribute to reduced thermotolerance in the respective plants, suggesting negative regulatory roles in heat stress responses for these HSBPs. In the present study, lines with overexpression of *BrHSBP1* were not found to differ phenotypically from wild-type lines in terms of their heat stress responses, possibly indicating differential roles for HSBPs of *B. rapa.* Additionally, overexpression of *BrHSBP1* resulted in larger seeds and pods compared to controls. Nevertheless, all of the loss-of-function mutants exhibited significant seed abortion to varying degrees. Unlike Arabidopsis, where mutants have been found to exhibit 33% seed abortion (Hsu et al., 2010), complete seed abortion was observed in T_0_ phenotypes of *Brhsbp1-KO* in this study. It is worth mentioning that the role of BrHSBP1-like is likely different from that of BrHSBP1, as differentiation in tissue preferences was observed, similar to the maize orthologs. It is clear that the BrHSBP1 overexpression and loss of function in *B. rapa* produced contrasting roles in seed and pod development, thus confirming the biological significance of this gene in these aspects of development.

To elucidate the molecular basis of BrHSBP1-mediated seed development, we selected *Brhsbp1-KO* heterozygous mutant seeds, due to the complete seed abortion observed in homozygous mutants. Genome-wide comparison between BrHSBP1^OX^ and CT revealed that *BrHSBP1* overexpression upregulates genes associated with developmental processes, to which the increased seed and pod size can be attributed. In general, plant genes that control the expression of developmental process-related genes can influence seed size (Savadi, 2018). In the stress response category, genes related to water deprivation-response genes and circadian rhythmic genes were mostly downregulated, while the broad category of abiotic stress response genes showed relative enhancement. It is worth to noting that preliminary drought phenotyping of BrHSBP1^OX^ during the vegetative growth phase showed enhanced drought tolerance compared to CT. Additionally, metabolic processes, particularly carbohydrate metabolic process, were upregulated in BrHSBP1^OX^ libraries. A genome-wide study in common bean cultivar has shown that induction of the carbohydrate metabolic process may be positively correlated with drought tolerance (Gregorio Jorge et al., 2020). A comparison between CT and *Brhsbp1-KO* showed contrasting results, with more genes being downregulated than upregulated, indicating that loss of function triggers relatively greater transcriptional reprogramming. Furthermore, developmental process genes, specifically those related to reproductive structure development, were largely reduced, corresponding to the seedless phenotypes observed in *Brhsbp1-KO* lines. It is evident from the literature that a reduction in the expression of reproductive structure development-related genes can directly or indirectly lead to defective seed development or seed abortion (Yang et al., 2003; Castillejo et al., 2005; Dong et al., 2005; Zhu et al., 2015; Huang et al., 2016; Yu et al., 2020). The same expression pattern was also reflected in a comparative analysis between BrHSBP1^OX^ and *Brhsbp1-KO* libraries. Moreover, the expression of ABA response genes showed a declining trend, while stress response genes did not show decisive differences in their regulation in mutants, despite the varying degree of magnitude in expression level. Among the upregulated processes in *Brhsbp1-KO*, signal transduction and gene expression regulation processes were notable. Taking the findings together, it can be concluded that BrHSBP1 is crucial for reproductive structure and shoot development in *B. rapa*, in addition to its role in stress responses.

To learn more about the mode of BrHSBP1 regulation over plant reproductive structure development (RSD) genes, we analyzed the possible interaction of BrHSBP1 with RSD genes using a yeast two-hybrid (Y2H) assay. Protein–protein interaction assays confirmed that BrHSBP1 directly binds to these genes, thus possibly modulating their expression. Among the candidate genes, *bHLH010* (BraA05g013660), a homolog in Arabidopsis, has been shown to be highly expressed in the tapetum of the anther. Mutants with respect to *AtbHLH010* result in defective anther phenotypes or aborted pollen development (Zhu et al., 2015). Therefore, we hypothesize that downregulation of BraA05g013660 in *Brhsbp1-KO* lines affects male fertility. Similar to *AtSPL8* (Yu et al., 2020), BraA09g068100 (squamosa promoter-binding-like protein 8) is likely to be involved in pollen sac development, gibberellin signaling, and male fertility. Similarly, BraA04g030840 (*SPL9*) is likely to control the juvenile-to-adult phase transition. *AtSPL9* plays additional roles in controlling trichome distribution and sesquiterpene and anthocyanin biosynthesis (Yu et al., 2020). In contrast, SEP3, which is a K-box region and MADS-box transcription factor family protein, has been shown to regulate inflorescence development and floral organogenesis (Pelaz et al., 2000; Honma and Goto, 2001). SEP3 is required for proper development of petals (Castillejo et al., 2005). ARPN (BraA02g037040) is expressed most highly in the transmitting tract of the pistil. The pistil, composed of the stigma, style, and ovary, is the female receptive organ in pollination, through which the pollen tube travels to deliver the sperm cells to the egg (Dong et al., 2005). ARPN has also been found to be highly expressed in mature seeds, and it is necessary to maintain the PIF1-mediated seed transcriptome and the low-GA-high-ABA state in Arabidopsis (Jiang et al., 2021). TAPETUM DETERMINANT1 (TPD1) (BraA01g015750) is required for cell differentiation in the anther and pollen development. Loss of function of TPD1 causes the precursors of tapetal cells to differentiate and develop into microsporocytes instead of tapetum (Yang et al., 2003; Huang et al., 2016). In Arabidopsis, TPD1 is reported to be expressed in flower buds, leaves, and young seedlings. In anthers, TPD1 is expressed throughout pollen development in parietal cells and sporocytes (Huang et al., 2016). AGL15 (BraA09g026780) (AGAMOUS-like 15) is a MADS-domain transcription factor (TF) that accumulates to its highest levels during embryogenesis, and it has been hypothesized that AGL15 participates in the regulation of embryo development (Perry et al., 1999). Hence, it can be reasoned that the loss of function of BrHSBP1 might negatively regulate RSD genes, resulting in seed- and pod-less phenotypes.

In general, seed development is accomplished through the concerted regulation of a complex network consisting of several signals, phytohormones, and genes (Kozaki and Aoyanagi, 2022). Of these, gibberellin is known to regulate seed development. In this study, gibberellin oxidase family genes were predominantly found to be differentially expressed in *Brhsbp1_KO* lines. Of these, *GA2ox2* [Bra032354], *GA20ox3* [Bra010064], *GA3ox1* [Bra026757], *GASA9* [Bra024530] and *GA2ox6* [Bra030500] were relatively predominant in flowers. This expression pattern suggests their possible contribution to seed development in *B. rapa*. On the other hand, expression of *GASA6* [Bra008162] was relatively higher in stem tissues. Previous studies have shown that enhanced expression of *GA2ox2* and *GA2ox6* reduces the active level of GAs, which ultimately leads to GA-deficient phenotypes (Li et al., 2019). In another study, it was proved that enhanced expression of *GA20ox* leads to early flowering and 25% greater height at maturity in Arabidopsis via improvement in endogenous GA levels (Huang et al., 1998; Coles et al., 1999). In general, GA2ox family genes in *A. thaliana* deactivate C_20_-GAs, and their overexpression in Arabidopsis and rice (*Oryza sativa*) results in GA-deficient phenotypes, including dwarfism (Li et al., 2019). Therefore, we presume that the induced expression in mutants of *GA2ox2*, *GA2ox6* and *GA2ox8*, and reduced expression of *GA20ox3*, is likely responsible for the appearance of GA-deficient *B. rapa* mutant phenotypes.

In addition to these findings, it is evident that BrHSBP1 can also bind to some HSR genes. While this study did not reveal distinguishable heat stress-responsive phenotypes in BrHSBP1^OX^ or *Brhsbp1-KO* lines (data not shown), HSR genes can influence other abiotic stress responses, either directly or indirectly, including drought stress, as described previously (Augustine et al., 2015; Wang et al., 2020). Further investigation is essential to establish the role of BrHSBP–HSF interactomes in drought tolerance in BrHSBP1^OX^ lines. One noteworthy difference between BrHSBP1^OX^ and CT is that a greater number of water-deprivation response genes were found to be downregulated compared to those that were upregulated as a whole. Notably, the largest number of reduced genes associated with water-deprivation response was also observed in *Brhsbp1-KO* lines. At this stage, further investigation is required to determine whether this expression pattern contributes to drought tolerance in BrHSBP1^OX^ lines. Additionally, during our preliminary studies, raffinose biosynthesis-related genes were found to exhibit significant expression changes in drought-stressed BrHSBP1^OX^ lines, which would be expected to contribute to drought tolerance (Li et al., 2020). Moreover, Y2H assay confirmed that BrHSBP1 interacts with BrGolS7, thereby supporting the role of BrHSBP1 in raffinose biosynthesis in *B. rapa* during drought stress conditions.

## 5 Conclusion

BrHSBP1 modulates the expression of genes related to plant reproductive structure development genes; this is directly or indirectly crucial for seed and pod development in *B. rapa*. In terms of its mode of action, BrHSBP1 can physically interact with target genes, as shown in protein–protein interaction studies. Furthermore, it is clear that BrHSBP1 can also regulate abiotic stress responses, including drought stress. An additional study detailing the molecular basis of drought tolerance in transgenic BrHSBP1 overexpression lines would provide additional knowledge about their biological significance in drought stress responses. Moreover, the modulation of gibberellin-related genes by BrHSBP1 and its involvement in drought stress response highlight its biological significance in adaptation to stress. Further unraveling of the molecular basis of HSBP1 in development and stress responses will present exciting opportunities for Brassica improvement programs aiming to enhance yield and stress tolerance.

## Data availability statement

The datasets presented in this study can be found in online repositories. The names of the repository/repositories and accession number(s) can be found in the article/**Supplementary Material**.

## Author contributions

MM and SL worked on conceptualization of the study, investigation, data analysis, and validation. MM worked on the writing of the original draft. SS and SP worked on review and editing of the manuscript. SL worked on fund acquisition and on project management and supervision. All authors contributed to the article and approved the submitted manuscript.
