# Supplementary figures and images for: Heat shock factor binding protein BrHSBP1 regulates seed and pod development in Brassica rapa
Source: Front Plant Sci. 2023 Aug 30;14:1232736. doi: 10.3389/fpls.2023.1232736 (PMC10499616; doi:10.3389/fpls.2023.1232736)

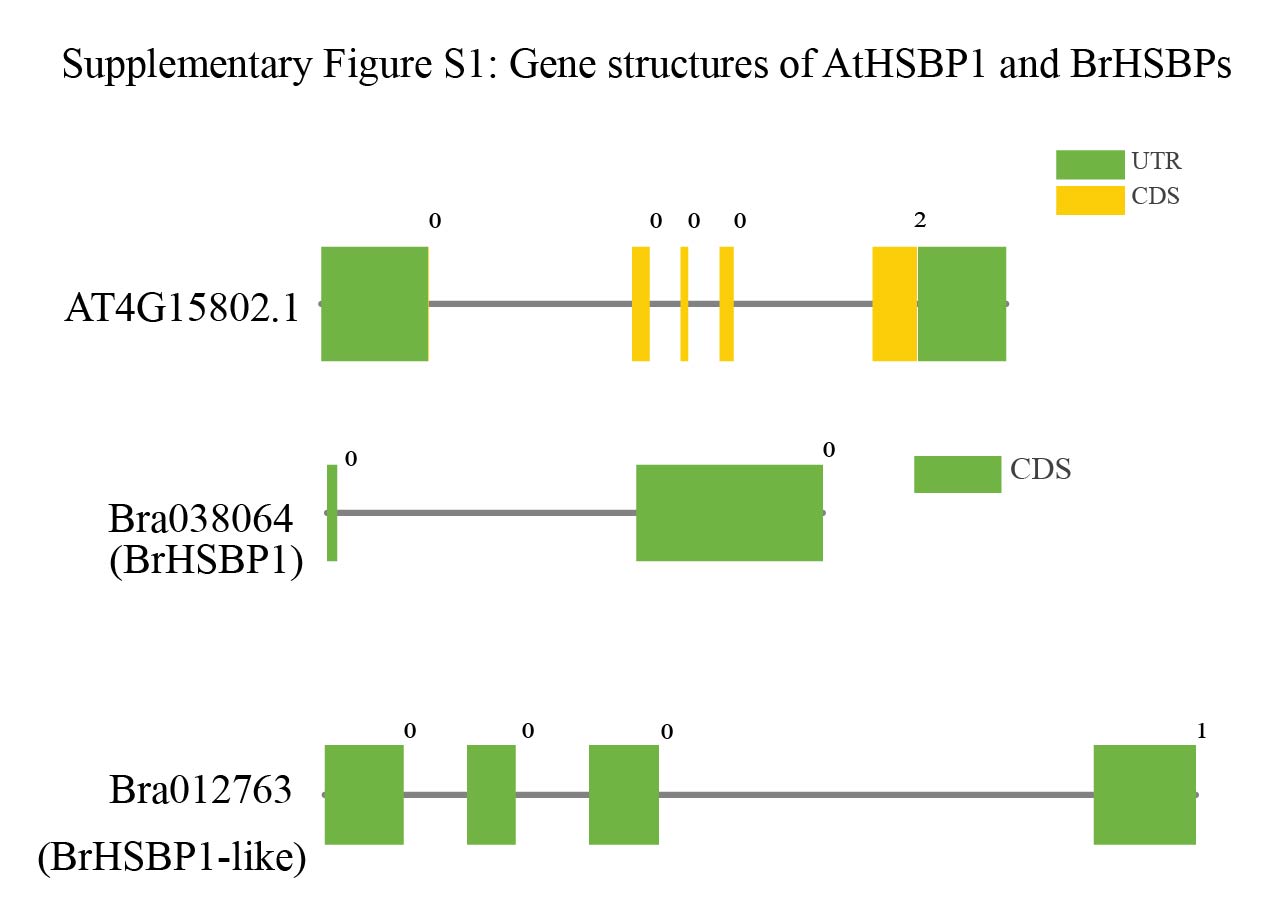

Supplement: Supplementary file 4 [file Image_1.jpeg]

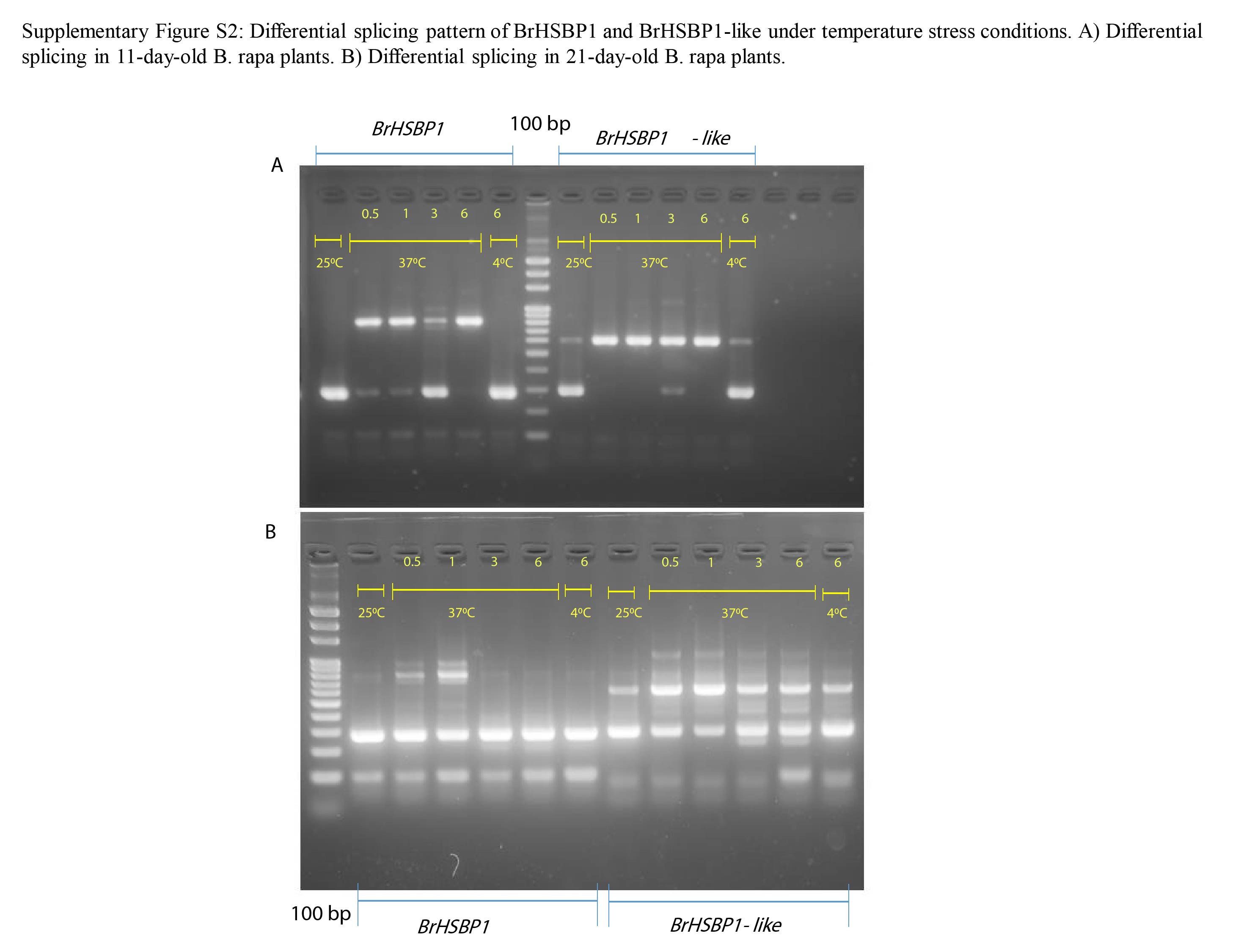

Supplement: Supplementary file 5 [file Image_2.jpeg]

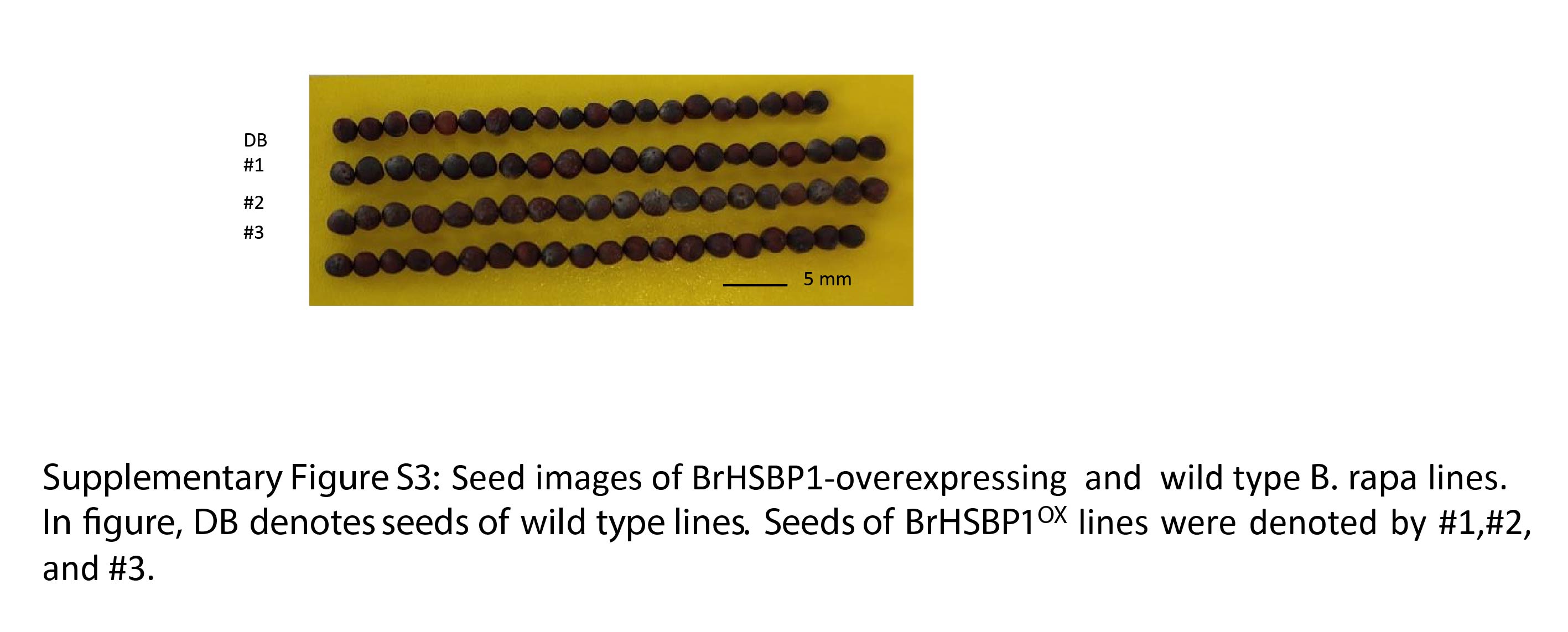

Supplement: Supplementary file 6 [file Image_3.jpeg]

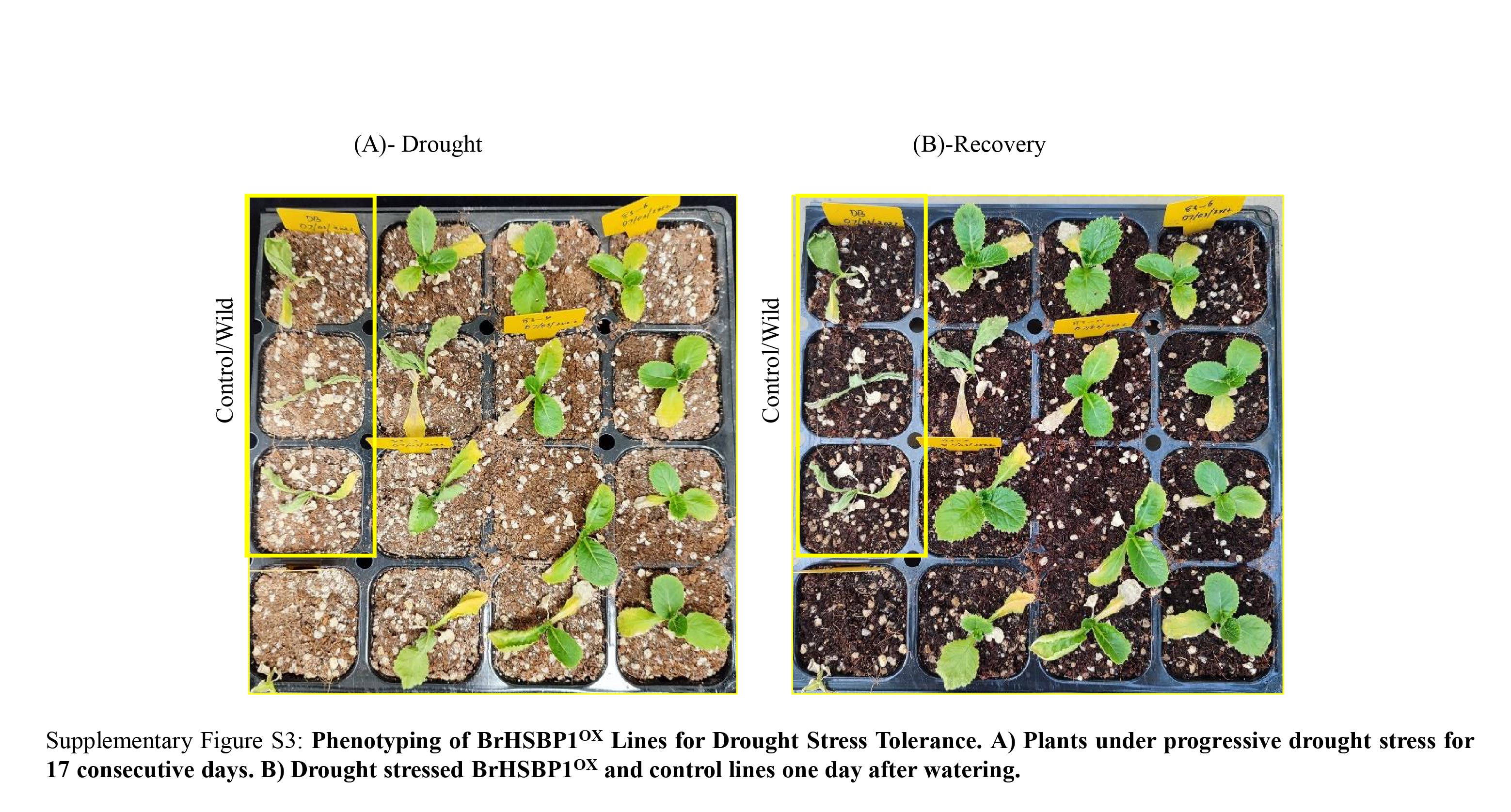

Supplement: Supplementary file 7 [file Image_4.jpeg]
