# Supplementary material for: Heat shock factor binding protein BrHSBP1 regulates seed and pod development in Brassica rapa
Source: Front Plant Sci. 2023 Aug 30;14:1232736. doi: 10.3389/fpls.2023.1232736 (PMC10499616; doi:10.3389/fpls.2023.1232736)
Supplement: Supplementary file 8 [file Table_1.docx]

Supplementary Material

Heat shock factor binding protein *BrHSBP1* regulates seed and pod development in *Brassica rapa*.

Muthusamy Muthusamy, Seungmin Son, Sang Ryeol Park, Soo In Lee*

*** Correspondence:**[silee@korea.kr](mailto:silee@korea.kr)

# Supplementary Table S1: Primer sequences of Raffinose biosynthesis pathway-related genes in *Brassica rapa*.

| **Primer Name** | **Sequence (5'->3')** | **Length** |
| --- | --- | --- |
| Bra025579-F | GCGAGAAATATGGCGGGAGA | 20 |
| Bra025579-R | AGATGGCTTCGGTTCCAAGG | 20 |
| Bra004474-F | TCAAGTACGTGACTGCACCG | 20 |
| Bra004474-R | GAGAGCGTGTGGTTGTCGTA | 20 |
| Bra027922-F | GTGGGTCTTGCTAAAGGGCT | 20 |
| Bra027922-R | TAAGCCATGGCAAACTCGGT | 20 |
| Bra031509-F | TACCCGGACCGTCAGTTACA | 20 |
| Bra031509-R | TCAAAAACAAGCCAAATCACACCT | 24 |
| Bra032505-F | CCAGTGTTGTCAATTGCGGT | 20 |
| Bra032505-R | AGGCTAAGACTCACACTTCGTT | 22 |
| Bra027156-F | TTTGAAACGCATTGTTCTGTTTCT | 24 |
| Bra027156-R | GAAACTTCCCATATACCGCCTC | 22 |
| Bra007842-F | GGTGATTGCCTTACCAGCCT | 20 |
| Bra007842-R | AAAGCAAGGAGGGTGATCGG | 20 |
| Bra030839-F | GTGGAGTACCGGTGTGATGG | 20 |
| Bra030839-R | CGAGCTTGCTCTTGTCTACGA | 21 |
