# Supplementary material for: Heat shock factor binding protein BrHSBP1 regulates seed and pod development in Brassica rapa
Source: Front Plant Sci. 2023 Aug 30;14:1232736. doi: 10.3389/fpls.2023.1232736 (PMC10499616; doi:10.3389/fpls.2023.1232736)
Supplement: Supplementary file 9 [file Table_2.docx]

Supplementary Material

Heat shock factor binding protein *BrHSBP1* regulates seed and pod development in *Brassica rapa*.

Muthusamy Muthusamy, Seungmin Son, Sang Ryeol Park, Soo In Lee*

*** Correspondence:**[silee@korea.kr](mailto:silee@korea.kr)

# Supplementary Table S2: List of Primer sequences used for cloning of BrHSBP1 and its potential interacting genes in mentioned Y2H vectors.

| **Primer Name** | **Seq (5`-3`)** | **Length** |
| --- | --- | --- |
| BraA09g026780-F | TAAGCACATATGATGTCTTATATGGTTTGTAGACAGCT | 38 |
| BraA09g026780-R | TGCTTAGGATCCTTAATTACATGAATGAAATTTGGAGGT | 39 |
| BraA07g012730-F | TAAGCACATATGAGAGAAGGAAAGAAGAGGAGAATGG | 37 |
| BraA07g012730-R | TGCTTAGGATCCTCAAATAGAGCTGGTGTCATAAGGT | 37 |
| BraA05g013660-F | TAAGCACATATGATGGAGAATCTAAATACTGATTTTGT | 38 |
| BraA05g013660-R | TGCTTAGGATCCTCAGTAGCCGTTGGCTGGAA | 32 |
| BraA02g037040-F | TAAGCACATATGCCCTCATTCATCATCTACAATCT | 35 |
| BraA02g037040-R | TGCTTAGGATCCTCAAACGGCCGTGACTGCTA | 32 |
| BraA09g068100-F | TAAGCACATATGATGTTGGACTACGAATGGGACA | 34 |
| BraA09g068100-R | TGCTTACCCGGGTTAAAGATTATACGCAGGAGCTAAGC | 38 |
| BraA01g015750-F | TAAGCACATATGATGAGCCGACGGCGACTTC | 31 |
| BraA01g015750-R | TGCTTAGAATTCTCAAGAGCAGGTAACAAACGC | 33 |
| HSFA1d1-FP | TAAGCAGAATTCATGGATCGCGGTAACAGAAC | 32 |
| HSFA1d1-RP | TGCTTACCCGGGTTACCTTGAAAGATCTATGGTTTCA | 37 |
| HSFA1b-2-FP | TAAGCACATATGATGGAATCGGTTCAATCCGCA | 33 |
| HSFA1b-2-RP | TGCTTAGGATCCTCATTTCCTCTGTGCTTCTGAAG | 35 |
| HSFA1e1-FP | TAAGCACATATGATGGGATCGAACAGCGAATCTGT | 35 |
| HSFA1e1-RP | TGCTTAGAATTCTCACTTCCTGAGCGCATCTGA | 33 |
| HSFA1d-2-FP | TAAGCACATATGATGGCTGGTGGTAATAGAAGCAG | 35 |
| HSFA1d-2-RP | TGCTTAGGATCCTTAGCCACTCTTCACTCCTCTG | 34 |
| HSFA1a-FP | TAAGCAGAATTCATGATGGATGGTGTAACCGCC | 33 |
| HSFA1a-RP | TGCTTACCCGGGTCACAGTTGCTTTGTCTCTGAAGTG | 37 |
| GolS7-FP | TAAGCAGAATTCATGATTTACTTAGATGCAGACATAC | 37 |
| GolS7-RP | TGCTTACCCGGGTCAAGCGGCGGAAGGTGC | 30 |
| BrHSBP1-FP | TAAGCACATATGATGGATGGGCATGATTCTGAGG | 34 |
| BrHSBP1-RP | TGCTTAGGATCCTCAAGTGGAATCAGCGGG | 30 |
| BrHSBP1-like-FP | TAAGCACATATGATGGATGGGCATGATTCTGAGG | 34 |
| BrHSBP1-like-RP | TGCTTAGGATCCCTAACTAGCCGGTGTTTTGGG | 33 |
